# Up to 70 THz bandwidth from an implanted Ge photoconductive antenna excited by a femtosecond Er:fibre laser

**DOI:** 10.1038/s41377-020-0265-4

**Published:** 2020-03-03

**Authors:** Abhishek Singh, Alexej Pashkin, Stephan Winnerl, Malte Welsch, Cornelius Beckh, Philipp Sulzer, Alfred Leitenstorfer, Manfred Helm, Harald Schneider

**Affiliations:** 1 Institute of Ion Beam Physics and Materials Research, Helmholtz-Zentrum Dresden-Rossendorf, 01328 Dresden, Germany; 2 Cfaed and Institute of Applied Physics, TU Dresden, 01062 Dresden, Germany; 3 Department of Physics and Center for Applied Photonics, University of Konstanz, 78457 Konstanz, Germany

**Keywords:** Infrared spectroscopy, Ultrafast photonics, Optoelectronic devices and components, Mid-infrared photonics, Terahertz optics

## Abstract

Phase-stable electromagnetic pulses in the THz frequency range offer several unique capabilities in time-resolved spectroscopy. However, the diversity of their application is limited by the covered spectral bandwidth. In particular, the upper frequency limit of photoconductive emitters - the most widespread technique in THz spectroscopy – reaches only up to 7 THz in the regular transmission mode due to absorption by infrared-active optical phonons. Here, we present ultrabroadband (extending up to 70 THz) THz emission from an Au-implanted Ge emitter that is compatible with mode-locked fibre lasers operating at wavelengths of 1.1 and 1.55 μm with pulse repetition rates of 10 and 20 MHz, respectively. This result opens up the possibility for the development of compact THz photonic devices operating up to multi-THz frequencies that are compatible with Si CMOS technology.

## Doping germanium for more terahertz

A negligible absorption of far-infrared radiation together with high electron mobility makes germanium a perfect material for photoconductive terahertz emitters. However, a very long ‘lifetime’ of photoexcited carriers is a serious disadvantage – electrons and holes excited by a laser pulse do not vanish until the next pulse arrives, they accumulate leading to a dramatic device degradation. The research team at the Helmholtz-Zentrum Dresden-Rossendorf in Germany has managed to overcome this limitation by introducing gold impurities into germanium, thus reducing the carrier lifetime by more than thousand times - down to the sub-nanosecond level. In cooperation with colleagues at the University of Konstanz (Germany) they demonstrated a germanium-based photoconductive antenna emitting terahertz pulses of “unprecedented” bandwidth of 70 THz using a femtosecond fibre laser. Further work on germanium-based THz devices is expected to revolutionize terahertz technologies, with applications in security, communication, and pharmaceuticals.

## Introduction

THz time-domain spectroscopy using broadband THz pulses has emerged as a powerful tool for probing low-energy excitations in condensed matter at the meV energy scale^1–3^. The spectrum of potential applications depends on the available spectral bandwidth, signal-to-noise ratio and data acquisition speed. In general, the techniques for THz generation and detection exploit either photoconductivity or optical nonlinearity^4,5^. Photoconductive techniques for THz emission and detection are widely used due to their simplicity, compactness and possibility of direct coupling to fiber optics. THz emission from photoconductivity was first demonstrated using Si^4,6,7^; however, the majority of current photoconductive antennas are based on GaAs or InGaAs (in case of the telecom wavelength) due to the high carrier mobility in these materials and well-established schemes for reducing the carrier lifetime^8^. Optical rectification techniques rely mostly on polar noncentrosymmetric materials with a strong second-order optical nonlinearity, such as ZnTe, GaP, GaSe, or *DSTMS*^9^. The polar nature of these materials renders their optical phonons strongly IR-active, leading to reststrahlen bands in the region between 5 and 10 THz. As a result, the spectral bandwidth of many THz emitters is limited to below 7 THz in the regular transmission mode. In particular, for InGaAs-based photoconductive emitters excited at a wavelength of 1.55 µm, gapless THz spectra up to 6.5 THz have been demonstrated^10^. Thin electro-optic crystals of GaSe and *DAST* have shown THz emission extending up to more than 100 THz towards the higher frequency end, but the THz intensity near their phonon frequencies is strongly suppressed^11–14^. Even in the reflection geometry available with photoconductive emitters, strong absorption and emission by polar TO and LO phonons, respectively, hinders their application for spectroscopy around the resonance frequencies^15,16^.

To satisfy the demand of a gapless ultrabroadband spectrum, novel techniques such as two-color air plasma^17^ and spintronic THz emission^18^ have been introduced. THz emission from air plasma achieves a bandwidth of more than 100 THz, but this technique requires high pump-pulse energies of several 100 µJ or higher that can be achieved only by rather complex and expensive laser amplifiers^17,19^. Spintronic emitters have shown great potential as a gapless broadband emitter reaching a bandwidth up to 30 THz that is compatible with nJ laser pulses from conventional femtosecond oscillators^18^. Recently, their scalability for the generation of higher THz fields was also demonstrated^20^. A similar study by Wu et al.^21^ demonstrated efficient operation of such a THz emitter driven by a pump power as low as 0.15 mW. Nevertheless, THz generation using photoconductive antennas remains important for many applications due to the direct control of the THz field strength and polarity by an applied bias voltage. Moreover, specially designed electrode geometries enable the generation of radial or azimuthal THz polarizations^22^ and a fully controllable angle of the linear polarization^23,24^. However, until recently, the bandwidth coverage of photoconductive emitters has been limited by the abovementioned factors.

A breakthrough in the generation of a broadband THz spectrum beyond the reststrahlen band of III-V semiconductors was achieved recently by using a Ge-based photoconductive dipole antenna based on pure Ge^25^. This semiconductor has a direct interband absorption above 0.8 eV, which is very close to its indirect bandgap at 0.66 eV. The effective electron mass in the center of the Brillouin zone of Ge is fairly small, leading to a strong acceleration of photogenerated electrons and, correspondingly, to efficient THz emission. This property gives Ge a clear advantage over Si in applications for photoconductive THz devices. Moreover, the relatively small bandgap of Ge enables pumping with compact fiber lasers. Finally, Ge is known to be compatible with Si CMOS technology^26^; thus, it is attractive for integrated on-chip THz solutions for THz signal processing^27,28^.

The absence of polar phonons in Ge enabled the generation of a gapless THz spectrum spreading up to 13 THz, and it has been demonstrated that the bandwidth of a Ge-based THz emitter is limited only by the duration of the excitation and detection laser pulses and, therefore, can be potentially extended to much higher frequencies^25^. However, the ultimate performance can hardly be reached for intrinsic Ge due to the relatively long carrier lifetime of several μs caused by the indirect character of the bandgap. The repetition rate of the driving laser must be low enough (250 kHz or less) to ensure full recombination of the carriers between the pulses, limiting the choice to complex and expensive regenerative laser amplifiers for which a sub-30 fs pulse duration is cumbersome to achieve, and the full pulse energy usually cannot be exploited due to the saturation of the THz emission by screening effects.

## Results

To harness the full potential of Ge as a material for photoconductive THz emitters, we reduce the carrier lifetime down to the sub-nanosecond level by introducing deep traps via Au implantation. Although a shorter carrier lifetime is not an essential requirement for broadband THz emission, a sub-nanosecond lifetime ensures reliable operation of Ge:Au THz emitters at repetition rates up to a few hundred MHz, covering the specifications of most contemporary femtosecond oscillators. It is known that Au in Ge forms deep acceptor levels within the bandgap that possess large capture cross-sections and drastically reduce the carrier lifetime for very low doping concentrations^29,30^. Ge substrates were implanted with Au ions with an energy of 330 keV and doses of 5 × 10^13^ ions/cm^2^ and 2 × 10^13^ ions/cm^2^ followed by annealing at 900 °C for several hours to ensure a low homogeneous concentration of gold impurities near the surface of the Ge wafer. After annealing, the Au ions diffuse hundreds of µm deep inside the Ge substrate, resulting in a suitable doping density of approximately 10^15^ cm^−3^ (see Supplementary Information Fig. S1).

**Fig. 1:**
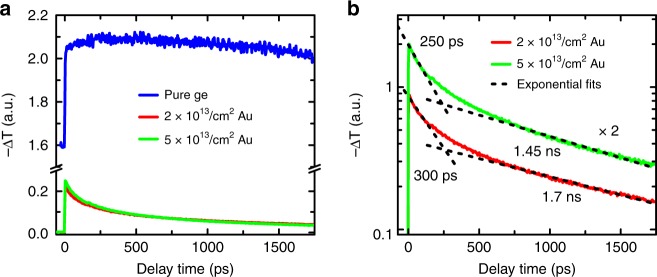
Carrier recombination in Ge measured by optical pump/THz probe spectroscopy. **a** Pump-induced change in the THz transmission as a function of the pump-probe delay time for pure and Au-implanted Ge. The pump fluence is 12.5 μJ/cm^2^, and the wavelength is 800 nm. **b** Bi-exponential fits of the decay dynamics for the Ge:Au samples. The curves for the 5 × 10^13^ ions/cm^2^ dose are vertically shifted for clarity (multiplied by 2)

We have estimated the carrier lifetime in implanted Ge wafers using optical pump/THz probe spectroscopy. Figure 1 shows the photoinduced change in the THz transmission Δ*T*, which is approximately proportional to the density of the free charge carriers. The comparison between pure and Au-doped Ge clearly demonstrates a dramatic reduction in the recombination time. The pure Ge sample shows a step-like increase in the carrier density with a minor decrease in the following 1.5 ns. Moreover, there is a strong nonzero response at negative delay times, indicating a high density of carriers accumulated in the sample from the preceding pump pulse arriving 4 μs earlier. In stark contrast, Ge:Au samples show a strong decay within 1 ns after photoexcitation and a negligible offset at negative delay times. The recombination dynamics can be described using a bi-exponential decay, as shown in Fig. 1b. The faster decay time of ≈300 ps can be attributed to surface recombination and the slower nanosecond decay to trap-assisted recombination in the volume of the Ge:Au sample. Both implanted Ge substrates exhibit a carrier lifetime of less than 2 ns, which is approximately 3 orders of magnitude shorter than the typical carrier lifetime in pure germanium.

**Fig. 2:**
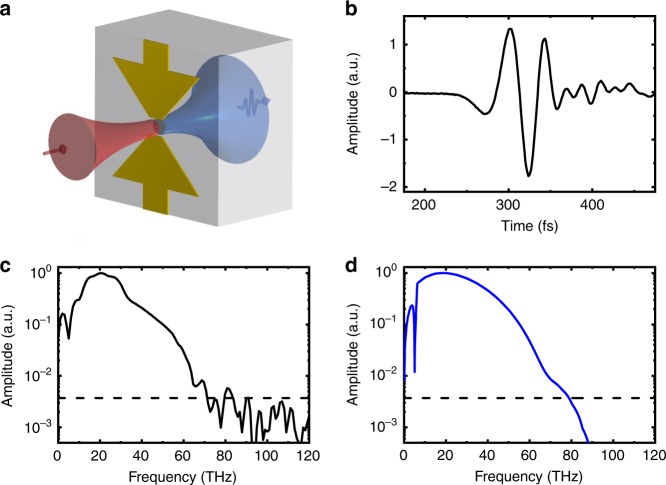
Ultrabroadband THz emission from a Ge:Au antenna pumped at 1100 nm. **a** Schematic diagram of the Ge photoconductive emitter with bowtie-like electrodes. The pump and THz beams are shown in red and blue, respectively. **b** Recorded THz pulse for pumping with 11-fs pulses with a wavelength centered at 1100 nm. **c** Fourier transform of the recorded THz pulse. **d** Simulated THz spectrum calculated as a product of the THz field emitted by the photoinduced current in the Ge emitter and the ZnTe detector response function. The dashed lines show the experimental noise floor

Bowtie electrode structures with a 10-µm gap and a 30-µm length for each electrode were fabricated on two implanted Ge:Au substrates using the same fabrication process and bowtie electrode geometry as in our previous work^25^. A schematic diagram showing THz emitter operation is presented in Fig. 2a. The near-infrared pump beam is focused onto the 10-µm gap between the electrodes. Photoexcited charge carriers are accelerated by the applied bias field, producing a transient current burst. The THz beam emitted by this current in the forward direction is collimated and refocused on an electro-optic crystal for field-resolved detection. The short carrier lifetime enables us to operate the Ge emitter at a repetition rate of tens of MHz using a femtosecond fiber laser system.

First, we test the THz emission induced by 11-fs short pulses with a central wavelength around 1100 nm (spectrum spanning from 900 to 1250 nm) and an energy of 7 nJ (at a repetition rate of 10 MHz). The emitter was fabricated on Ge:Au with an implantation dose of 2 × 10^13^ ions/cm^2^. The generated THz transient is detected by electro-optic sampling using 8.42-fs short probe pulses in a (110) ZnTe crystal with a thickness of 14.3 µm. Figure 2b, c show the recorded THz pulse in the time domain and its Fourier spectrum, respectively. The obtained spectrum spans from the lowest detectable frequency up to 70 THz, demonstrating unprecedented bandwidth for a photoconductive antenna with a gapless spectrum. The small dip in the spectrum at approximately 5 THz is caused by the response function of the ZnTe detector, and the true THz emission spectrum is gapless, as has been confirmed in previous work^25^. The estimated peak electric field of the focused THz pulse is ~ 0.8 kV/cm, and the signal-to-noise ratio is ~300.

We have modeled the detected THz spectrum using the intensity and phase spectra of the pump and probe near-infrared pulses (see Supplementary Information). The emission from biased Ge:Au is calculated by solving the equation for the pump pulse propagation in Ge, which takes into account the absorption and dispersion of the refractive index. In this way, the temporal and depth profile of the photoinduced carrier concentration can be calculated. The total emitted THz field is estimated as a sum of the emission by currents across the full depth of the emitter. The resulting spectrum is multiplied by the detector response function (DRF) of the ZnTe detector, which takes into account the group velocity dispersion across the very large bandwidth of the probe pulse. Further details are given in the Supplementary Information. The effect of the diffraction-limited focusing of the THz beam on the detector is also taken into account. The result shown in Fig. 2d agrees well with the experimentally measured spectrum demonstrating that the roll-off at the higher frequency is mainly due to widths of the pump and probe pulses, and the roll-off towards the lower frequency is due to diffraction-limited focusing of the THz beam on the detector crystal. Thus, we observe a gapless spectrum extending up to 70 THz (wavelength of 4.3 μm).

**Fig. 3:**
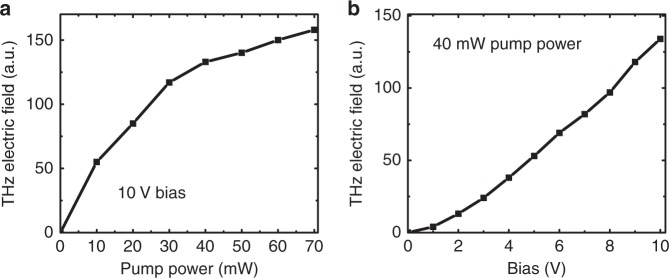
Peak-to-peak electric field of the THz pulse. **a** THz peak-to-peak electric field variation at a constant bias of 10 V and various pump powers. At a high pump-pulse energy, the separation of photogenerated electron-hole pairs causes screening of the DC field applied to the emitter. Hence, the efficiency of the emitter is affected, causing saturation of the emitted THz field. **b** THz peak-to-peak electric field variation at a constant pump power of 40 mW and a varying bias applied to the emitter electrodes

The performance of the implanted Ge emitter is studied for various pump powers and applied bias. Figure 3a, b show the observed variation in the peak-to-peak electric field amplitude of the emitted THz pulse at different pump powers and applied bias, respectively. Usually, the peak-to-peak electric field amplitude of photoconductive emitters increases linearly with the pump power until saturation occurs due to the screening of the applied electric field. The implanted Ge emitter also exhibits similar behavior with the pump power, as shown in Fig. 3a. The THz field scales almost linearly for a pump pulse energy up to 3 nJ (30 mW of power at 10 MHz), exhibiting saturation beyond this limit. Similarly, the THz field is also expected to scale linearly with the applied bias field on the emitter electrodes. Figure 3b demonstrates an almost linear dependence with the signal vanishing at zero bias, thus confirming that the THz emission is produced solely by the photoinduced current in the Ge antenna.

In the following, we demonstrate the compatibility of the Ge:Au photoconductive antenna with the conventional telecom C-band covered by ubiquitous Er-doped femtosecond fiber lasers. The absorption edge of Ge at 0.66 eV (*λ* ≈ 1876 nm) is well below the telecom band. However, the absorption is rather weak due to the indirect character of the bandgap and increases abruptly only for photon energies above 0.8 eV (*λ* ≈ 1550 nm) corresponding to direct interband transitions near the Γ-point of the Brillouin zone^31^. The onset of the strong absorption in Ge coincides with the telecom wavelength of 1550 nm and, thus, should enable broadband emission from the Ge-based THz emitter.

**Fig. 4:**
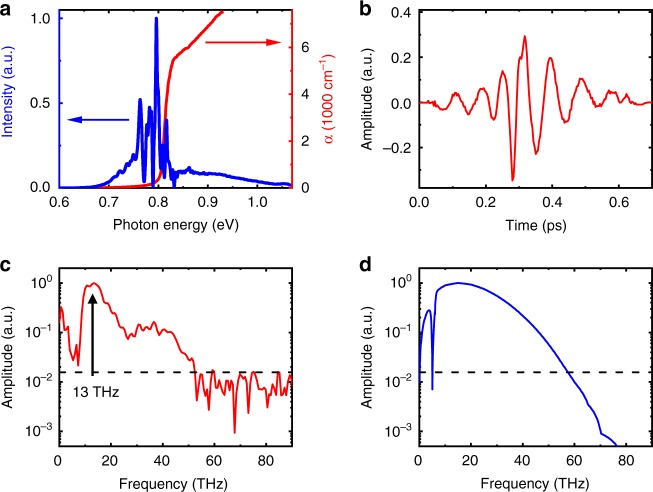
Broadband THz emission from the Ge:Au antenna pumped at 1550 nm. **a** Spectrum of the pump pulse (blue) compared to the absorption coefficient of Ge (red) taken from Ref. ^29^. **b** Recorded THz pulse for pumping with 12-fs laser pulses centered around 1550 nm. **c** Fourier transform of the recorded pulse. The arrow indicates the contribution of the narrowband part of the pulse at 13 THz. **d** Simulated THz spectrum. Only the broadband single-cycle pulse stems from the photocurrent. The dashed lines show the experimental noise floor

The emitter fabricated on Ge:Au with an implantation dose of 5 × 10^13^ ions/cm^2^ was pumped with 12-fs pulses with a central wavelength around 1550 nm and an energy of 3.5 nJ at a repetition rate of 20 MHz. The spectrum of the pump pulse is shown in Fig. 4a together with the absorption coefficient of Ge, demonstrating that the part of the pump pulse with photon energies below 0.8 eV should contribute much less to the generation of the transient photocurrent than its high-energy part. Thus, even though the initial pulse duration and the energy are comparable to the previous case of pumping at 1100 nm, the expected THz bandwidth should be lower than 70 THz.

The emitted THz pulses are recorded with an 18-µm-thick ZnTe detector crystal by electro-optic sampling using 5.8-fs pulses with a central wavelength of 1200 nm (see Supplementary Information). The photoconductive antenna is pumped at a repetition rate of 20 MHz under an applied DC bias of 10 V. Figure 4b, c show the recorded THz waveform and its Fourier transform, respectively. The THz spectrum spans up to 50 THz, demonstrating that the Ge-based photoconductive antenna is capable of broadband THz emission when pumped at the telecom wavelength. As anticipated, the bandwidth and dynamic range of the THz waveform are lower than those for pumping at 1100 nm due to the nonuniform absorption of different parts of the excitation spectrum. The estimated peak electric field is ~ 0.12 kV/cm, and the signal-to-noise ratio is ~65. Furthermore, the pulse shape differs from the typical single-cycle THz waveform that is observed for pumping at 1100 nm (see Fig. 2b) and includes an additional multi-cycle THz component with a frequency of approximately 13 THz. The phase of this narrowband feature is nearly opposite to that of the broadband THz pulse at low frequencies, leading to the minimum in the detected spectrum below the 13 THz peak due to destructive interference.

## Discussion

Although similar narrowband emission by LO phonons in polar semiconductors is well known and understood^15,32^, such a component is not expected in nonpolar Ge. To verify whether this emission can be related to indirect interband absorption away from the surface of the photoconductive antenna, we performed a simulation of the recorded THz pulse using the same approach as that for 1100-nm pumping. The time-dependent current across the full depth of the Ge:Au wafer is calculated by numerically solving the pulse propagation equation (see Supplementary Information). The resulting THz spectrum depicted in Fig. 4d reasonably describes the decrease in the bandwidth to 50 THz due to the dominating role played by the carriers photoexcited via the direct interband transitions for photon energies above 0.8 eV. In fact, our simulation shows a negligible contribution of the spectral components below 0.8 eV. However, the THz emission is expected to be similar to the case of pumping at 1100 nm with a nearly single-cycle THz waveform. Thus, the narrowband emission at 13 THz cannot be attributed to a standard transient photocurrent in our structure. A more exotic mechanism such as coherent polarizations due to the simultaneous generation of heavy-hole–light-hole wavepackets as recently reported in GaAs^33^ may be considered. However, this discussion is beyond the scope of the present work and requires a larger amount of experimental data.

In conclusion, we have demonstrated a photoconductive THz emitter fabricated on Au-implanted Ge that is capable of emitting a gapless spectrum with an unprecedented bandwidth reaching 70 THz. The tested devices are fully compatible with femtosecond fiber lasers and demonstrate ultrabroadband THz emission by pumping at either 1100 or 1550 nm. Thus, Ge-based THz emitters may be used for the generation of a gapless spectrum in combination with standard Er-doped femtosecond fiber lasers at frequencies as high as 76 MHz. The demonstrated bandwidth is almost one order of magnitude higher than that of existing state-of-the-art photoconductive THz emitters fabricated on GaAs or InGaAs. Consequently, Ge-based THz devices can revolutionize THz technology due to their ultrabroad spectral bandwidth coverage and their potential compatibility with Si CMOS technology.

## Materials and methods

### Ge implantation

Au ions with an energy of 330 keV and doses of 2 × 10^13^ ions/cm^2^ and 5 × 10^13^ ions/cm^2^, respectively, were implanted into two nominally undoped (100) Ge substrates. A simulation using the SRIM software shows an initial implantation depth of approximately 150 nm (Supplementary Information Fig. S1). To distribute the Au ions uniformly over a length scale larger than the penetration depth of the pump light and to recover the lattice damage after ion irradiation, the samples were annealed in vacuum at 900 °C for 3 h (sample with a dose of 5 × 10^13^ ions/cm^2^) and 10 h (sample with a dose of 2 × 10^13^ ions/cm^2^). Annealing is expected to cause Au diffusion to a depth of more than 100 µm into the Ge wafers. After processing, the surfaces of the samples were polished to make them smooth enough for lithographic processing.

### Optical pump/THz probe measurements

The measurements of the carrier lifetime in Ge:Au were performed using a THz setup equipped with a GaAs-based large-area photoconductive emitter and a ZnTe electro-optic detector covering frequencies up to 3 THz. The system is driven by a Ti:Sa amplifier laser operating at a repetition rate of 250 kHz and a wavelength of ~ 800 nm. Pump powers of 50 and 200 mW are used to pump the ≈3-mm-diameter area, resulting in fluences of 12.5 and 50 μJ/cm^2^ for the excitation of pure and implanted Ge substrates, respectively. The THz pulse is focused to approximately 1 mm in size on the same spot, ensuring homogeneous pumping conditions. The change in the THz transmission is measured using lock-in detection at different pump-probe delay times. The data shown in Fig. 1 are recorded at the peak of the THz probe pulse and normalized with respect to the pump fluence in order to provide a direct comparison of the different curves.

### Emitter fabrication

Bowtie-like electrodes were fabricated on the two implanted Ge substrates using standard electron beam lithography. Two layers of 5-nm Ti and 45-nm Au were deposited consecutively, and the bowtie geometry was formed by a lift-off process.

### THz emitter characterization for pumping at 1550 and 1100 nm

The THz setup is based on ultrabroadband Er:fiber laser technology^34^. The repetition rates of the electro-optic sampling pulses with wavelengths of 1100 and 1550 nm are 20 and 40 MHz, respectively. The pump pulses with wavelengths of 1100 or 1550 nm are modulated at 10 and 20 MHz, respectively—half of the oscillator frequency, enabling excellent sensitivity of the system at the shot-noise limit using a lock-in detection technique. Further details can be found in the additional materials of recent publications that utilized the same fiber laser systems^35,36^.

An 18-µm-thick ZnTe crystal was used for the electro-optic sampling of the emitted THz pulses when the emitter was pumped at a wavelength of 1550 nm, and 14.3-µm-thick ZnTe and 18.4-µm-thick GaSe were used to characterize the emitter when pumped at 1100 nm. The studied photoconductive emitters were biased by a static voltage, since the pump pulses were already modulated at half the probe repetition rate.

## Supplementary information

Supplemental Material
