# Supplementary material for: Up to 70 THz bandwidth from an implanted Ge photoconductive antenna excited by a femtosecond Er:fibre laser
Source: Light Sci Appl. 2020 Mar 3;9:30. doi: 10.1038/s41377-020-0265-4 (PMC7052201; doi:10.1038/s41377-020-0265-4)
Supplement: Supplementary file 1 — Supplemental Material [file 41377_2020_265_MOESM1_ESM.docx]

**Supplementary information**

**Up to 70 THz bandwidth from an implanted Ge photoconductive antenna excited by a femtosecond Er:fibre laser**

Abhishek Singh^1^, Alexej Pashkin^1*^, Stephan Winnerl^1^, Malte Welsch^1,2^, Cornelius Beckh^3^, Philipp Sulzer^3^, Alfred Leitenstorfer^3^, Manfred Helm^1,2^, and Harald Schneider^1*^

^1^ Institute of Ion Beam Physics and Materials Research, Helmholtz-Zentrum Dresden-Rossendorf, 01328 Dresden, Germany

^2^ Cfaed and Institute of Applied Physics, TU Dresden, 01062 Dresden, Germany

^3^ Department of Physics and Center for Applied Photonics, University of Konstanz, 78457 Konstanz, Germany

^*^ Corresponding Authors: [a.pashkin@hzdr.de](mailto:a.pashkin@hzdr.de) or [h.schneider@hzdr.de](mailto:h.schneider@hzdr.de)

**Au implantation and diffusion due to annealing**

The Au ion distribution in Ge wafer after the 330 keV ion energy implantation is simulated using the software SRIM and results are shown as insets in Figs. S1a and Fig. S1b for the doses of 2×10^13^ ions cm^‑2^  and 5×10^13^ ions cm^‑2^, respectively. After annealing at 900 ̊C ions diffuse inside the Ge wafer. The Au ion distribution in Ge after diffusion is calculated and results are shown in Figs. S1a and S1b. The diffusion coefficient of Au in Ge at 900 ̊C is taken from Ref. R1.

**
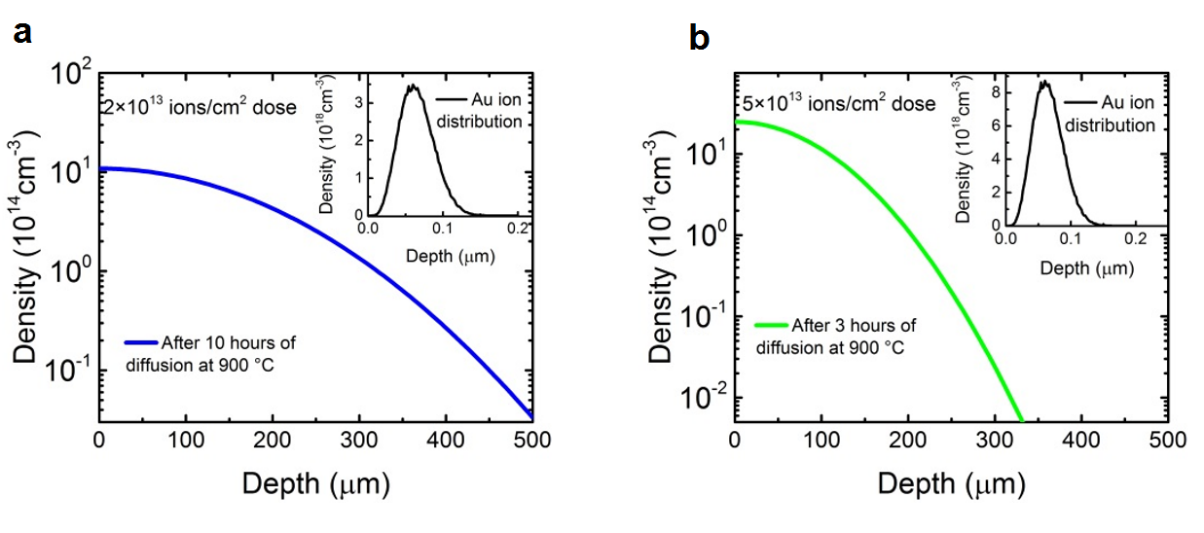
**

**Figure S1. Au concentration in Ge after ion irradiation and post anealing**. **a**, Ge:Au with the dose of 2x10^13^ cm^-2^ **b**, Ge:Au with the dose of 5x10^13^ cm^-2^. The insets show the distribution of the implanted Au ions before the annealing.

**Modelling of the photoinduced current**

In order to model the photocurrent we calculate the evolution of the near-infrared pump pulse during its propagation in the depth of the Ge:Au substrate. The temporal profile of the pump electric field at the given depth *z* is equal to

$$\boldsymbol{E}\left( \boldsymbol{z,t} \right)\boldsymbol{=}\int_{\boldsymbol{0}}^{\boldsymbol{\infty}} \boldsymbol{E}_{\boldsymbol{0}}\boldsymbol{(\omega)}\boldsymbol{e}^{\boldsymbol{-\omega\kappa(\omega)z/c}}\boldsymbol{e}^{\boldsymbol{i\omega(n(\omega)z/c-t)}}\boldsymbol{d\omega}\boldsymbol{,}$$

where *n^*^ = n*(*ω*) *+ iκ*(*ω*) is the complex refractive index of Ge and *E*_0_(*ω*) is the spectrum of the pump pulse including its phase. In the numerical modelling, the integration is taken over the full bandwidth of the pump pulse.

The photogenerated carrier density is estimated as

$$N\left( z,\tau\right)=\frac{\alpha(\omega)\Phi(z,\tau)}{\hbar\omega}$$

where *α* is the absorption coefficient and *Φ* is the pump fluence. The simulation results for both pump wavelengths are shown in Fig. S2. The time scale is given in the lab reference system, i.e., it is corrected with respect to the propagation velocity of the THz pulse. Thus, the temporal spreading of the photoexcited carriers in the presented plots results solely from the difference between the group velocity of the pump pulse and the phase velocity of the THz transient. One can clearly see that in spite of the comparable duration for the pump pulses at 1100 nm and 1550 nm of 11 fs and 12 fs, respectively, the carrier excitation spreads over noticeably longer time in the latter case.


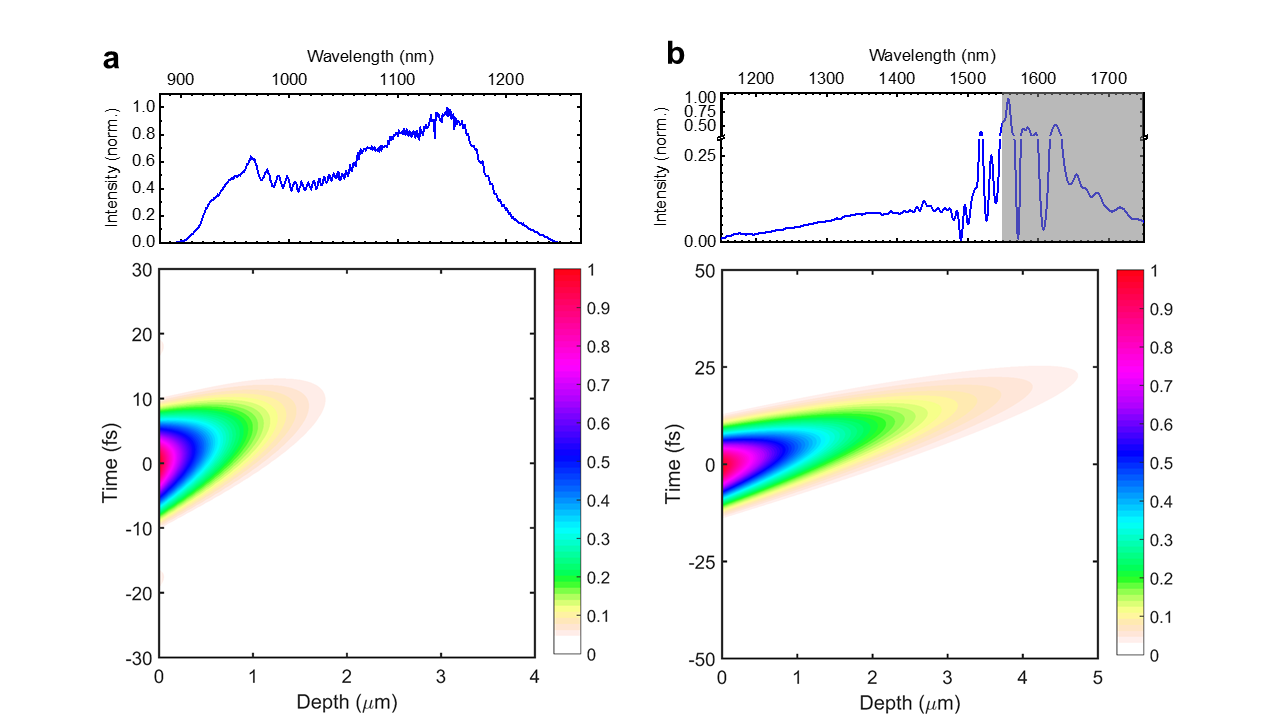


**Figure S2. Pump-induced carrier density *N*(*z*,*τ*) in the Ge:Au substrates as the function of the depth *z* and the time *τ*.** The time *τ* corresponds to the reference system of the electro-optic detector and takes into account the delay due to the propagation of the generated THz pulse through the substrate. **a**, Excitation at the center wavelength of 1100 nm; **b**, Excitation at the center wavelength of 1550 nm. Note the different scaling in the graphs. The upper panels show the respective spectra of the pump pulses.

As discussed in the main text, this is related to the very weak absorption in Ge below the edge of the direct interband absorption that corresponds to ≈ 1550 nm (see Fig. 4a). Thus, only the short-wavelength part of the spectrum contributes to the carrier generation resulting in the spectral narrowing and, consequently, in the temporal broadening of the photoinduced carrier distribution.

The transient photocurrent is calculated as a product of the carrier density and the bias electric field. Since the pump light penetrates for several micrometres into the substrate, it is necessary to know the spatial distribution of the applied dc electric field between the two electrodes of the emitter. We use COMSOL Multiphysics software to calculate the electric field distribution between the bowtie electrodes when a bias of 10 V is applied. The *y*-component of the electric field parallel to the THz polarization is shown in Fig. S3a for the *yz*-plane passing through the centre of the bowtie structure. The electric field variation in the depth along the dotted line in the middle of the electrode gap (see Fig. S3a) is depicted in Fig. S3b. The electric field drops rapidly up to 15 µm depth, but drops only slightly within the first 5 μm where most of charge carriers are generated. Definitely, the pumping at 1550 nm also generates carriers deeper in the substrate (beyond 5 μm depth) due to the absorption across the indirect bandgap, but according to our simulation their contribution to the photocurrent is negligibly small.

**
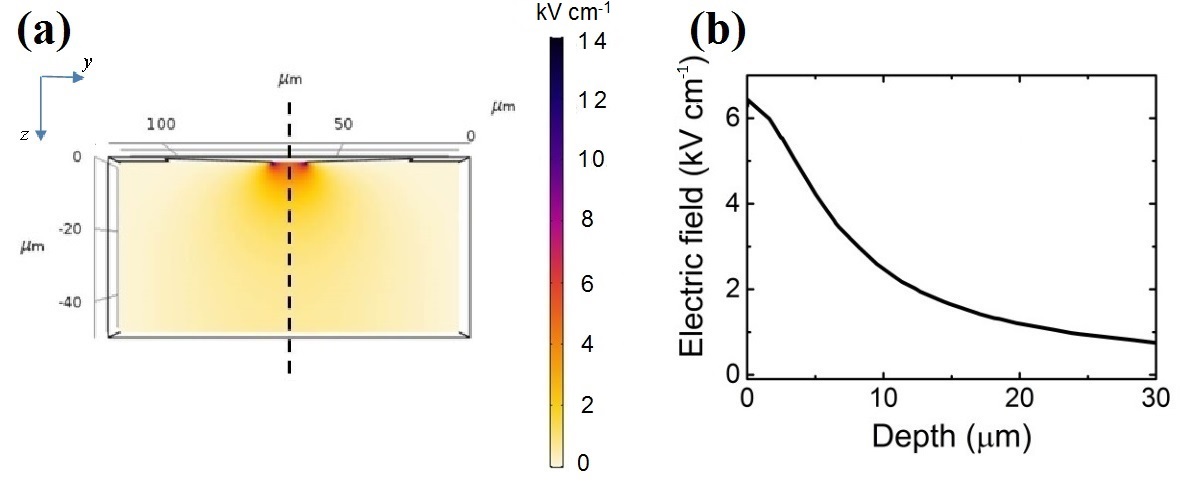
**

**Figure S3. Electric field distribution inside the Ge emitter.** **a**, *y*-component of electric field in a *yz*-plane passing through the center of the emitter; **b**, Electric field distribution along a line (dotted line in panel **a**) along the *z*-direction passing through the center of the emitter.

**Detector Response Function**

In order to calculate the detected THz field, the emitted THz radiation in the far field, which is proportional to the time derivative of the total photocurrent, has to be multiplied by the spectral response function of the electro-optic detector. The detector response functions of ZnTe detector crystals are calculated using the method given in reference 32. The response function $R\left( \omega\right)$ is a function of THz frequency (ω);

$$R\left( \omega\right)={G(\omega)\times r}_{e}(\omega)$$

where

$$G\left( \omega\right)=\frac{2}{n\left( \omega\right)+1}\times\frac{c\times[\exp\left\{ -{i2\pi\omega d\left( n_{g}\left( \lambda_{0} \right)-n\left( \omega\right) \right)}/c \right\}-1]}{-i2\pi\omega d\left( n_{g}\left( \lambda_{0} \right)-n\left( \omega\right) \right)}$$

$$n\left( \omega\right)=\sqrt{\left[ 1+\{\frac{\left( \hbar\omega_{LO} \right)^{2}-\left( \hbar\omega_{TO} \right)^{2}}{\left( \hbar\omega_{TO} \right)^{2}-\left( \hbar\omega\right)^{2}-i\hbar\gamma\omega}\} \right]\times\varepsilon_{\infty}}$$

and

$$r_{41}\left( \omega\right)=r_{e}\times[1+C{\{1-\frac{{(\hbar\omega)}^{2}-i\hbar\gamma\omega}{\left( \hbar\omega_{TO} \right)^{2}}\}}^{-1}]$$

For ZnTe, *ħω_LO_* = 6.18 THz; *ħω_TO_* = 5.3 THz; $\gamma$= 0.09 THz; *ε*_∞_ = 6.7; *C* = -0.07 (according to reference 32); *r_e_* is a constant chosen as 1 here; and $n_{g}\left( \lambda_{0} \right)$ is the group refractive index of ZnTe at the probe pulse wavelength (*λ_0_*). The probe pulse length and spectrum are shown in Fig. S4a&b and Fig. S5a&b for probe pulses used in setups with 1100 nm and 1550 nm pumping, respectively. Since the probe pulse is a broadband pulse and the group refractive index *n_g_*(λ_0_) varies across the probe pulse spectrum, the detector response function $R\left( \omega\right)$ will also have different values for different probe wavelengths. The detector response function (DRF) is calculated at 13 discrete values of the probe wavelength (shown with square dots in Figs. S4a and S5a. DRF at three such wavelengths are shown in Figs. S4c and S5c by red, green and blue curves, respectively. DRFs calculated at all discrete wavelengths are then multiplied by the probe intensity at the corresponding wavelengths (*λ_0_*) to get a weighted average of the DRF. Average DRFs are shown with black curves in Figs. S4d and S5d.


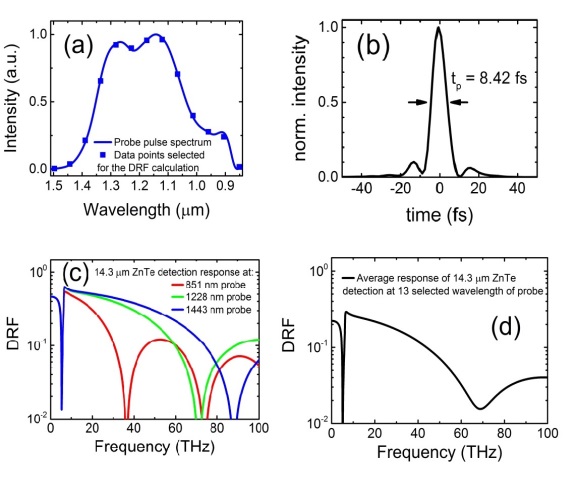


**Figure S4. Detector response function for the setup with 1100 nm pumping.** **a**, Spectrum of the probe pulse and chosen 13 data points for the calculation of DRF. **b**, Temporal profile of the probe pulse. **c**, DRFs at different probe wavelengths. **d**, Complete DRF obtained by weighted average of the DRFs across the probe spectrum.

**Frequency response due to pulse width used for electro-optic sampling**

The width of the pump pulse used to generate the charge carriers in the emitter is taken into account by our simulation of the transient photocurrent. On the other hand, the finite duration of the EOS probe pulse also limits the bandwidth of the recorded THz waveform. To determine the frequency roll-off due to the finite width of the probe pulse, the Fourier transform of the temporal pulse shape (shown in Figs. S4b and S5b) is multiplied with the DRF.

**Frequency response due to THz focusing on the detector**

At the detector crystal the NIR probe and THz pulse to be detected are focused as tightly as possible to get the maximum electro-optic signal. Since the focus spot size is limited by diffraction, smaller wavelengths are focused to smaller spot size. NIR probes have spot diameters much smaller than the THz spot diameter. The observed THz signal depends on THz electric field (square root of intensity) overlapping with the probe focus spot on the detector (actually overlapping volume in detector crystal). The THz intensity on the NIR probe focus spot is inversely proportional to the focus spot area of the THz wavelength; hence the observed THz signal is directly proportional to the THz frequency. This effect is included in the final simulation by multiplying the DRF with the corresponding THz frequency (*ω*) and the Fourier transform of the probe pulse as mentioned above.


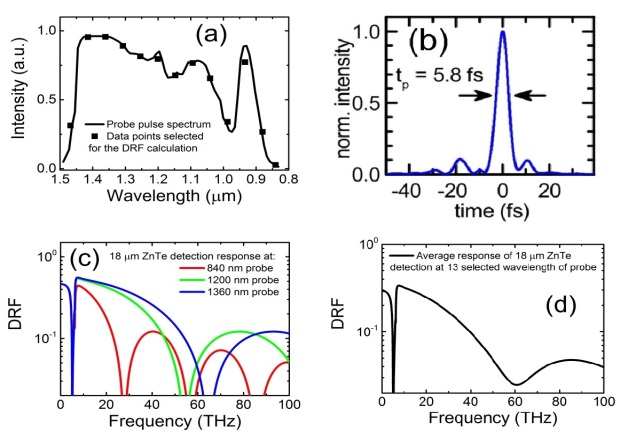


**Figure S5. Detector response function for the setup with 1550 nm pumping. a**, Spectrum of the probe pulse and chosen 13 data points for the calculation of the DRF. **b**, Temporal profile of the probe pulse. **c**, DRFs at different probe wavelengths. **d**, Complete DRF obtained by weighted average of the DRFs across the probe spectrum.

**70 THz bandwidth with GaSe detector**

The THz signal emitted from the Au-implanted (2×10^13^ ions cm^-2^) Ge-based photoconductive THz emitter, when pumped with 1100 nm, is also detected with an 18.4 µm GaSe electro optic crystal. Like the results shown in Fig. 4(b) in the main article using a ZnTe detector, a THz spectrum up to 70 THz is recorded as shown in Fig. S6.


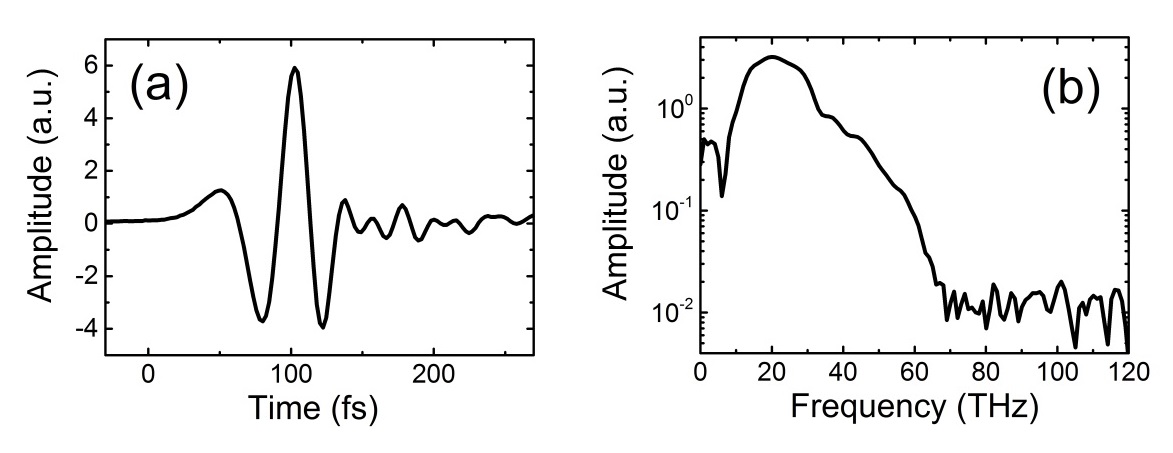


**Figure S6.** (a) THz pulse recorded using GaSe detector and (b) corresponding spectrum. A spectral bandwidth up to 70 THz is achieved.

**Emitter compatibility with 76 MHz repetition rate pumping**

To demonstrate the compatibility of implanted Ge emitters with a repetition rate even higher than 20 MHz, we tested the emitter with dose 5 × 10^13^ ions cm^-2^ using a conventional Ti:Sapphire oscillator operating at the wavelength of 800 nm and the repetition rate of 76 MHz. The pulse duration is nearly 100 fs and a 1-mm-thick <110> ZnTe crystal is used as electro-optic detector. 100 mW pump power and 10 V (= 10 kV cm^-1^ electric field) bias are used on the emitter. The recorded time-domain pulse and its Fourier transform are shown in Fig. S7. The high-frequency cut off is below 4 THz due to the thickness of the ZnTe detector and the laser pulse width used for generation and detection of the THz pulses. However, the performance of the Ge:Au antenna is comparable to its GaAs-based analogue in agreement with our previous study.^25^


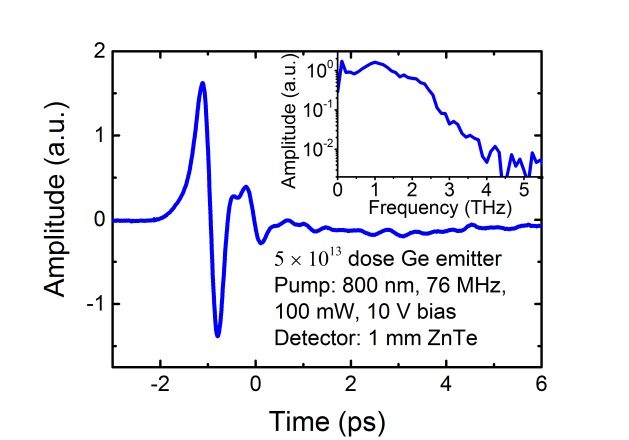


**Figure S7. Emitter performance with 76 MHz, 100 fs, 800 nm.** THz pulse emitted from implanted Ge emitters when pumped with ~ 100 fs, 800 nm Ti:Sa laser pulses at 76 MHz. The spectral bandwidth is limited due to the pulse width of pump and probe pulses, and the thickness of the ZnTe detector crystal.

**References**

[R1] Bracht, H., Stolwijk, N. A. & Mehrer, H. Diffusion and solubility of copper, silver, and gold in germanium. *Phys. Rev. B* **43**, 14465-14477 (1991).
